# Supplementary material for: Complex‐centric proteome profiling by SEC‐SWATH‐MS
Source: Mol Syst Biol. 2019 Jan 14;15(1):e8438. doi: 10.15252/msb.20188438 (PMC6346213; doi:10.15252/msb.20188438)
Supplement: Supplementary file 7 — Dataset EV6 [file MSB-15-e8438-s007.zip › feature_plots_bioplex/Q99848.pdf]

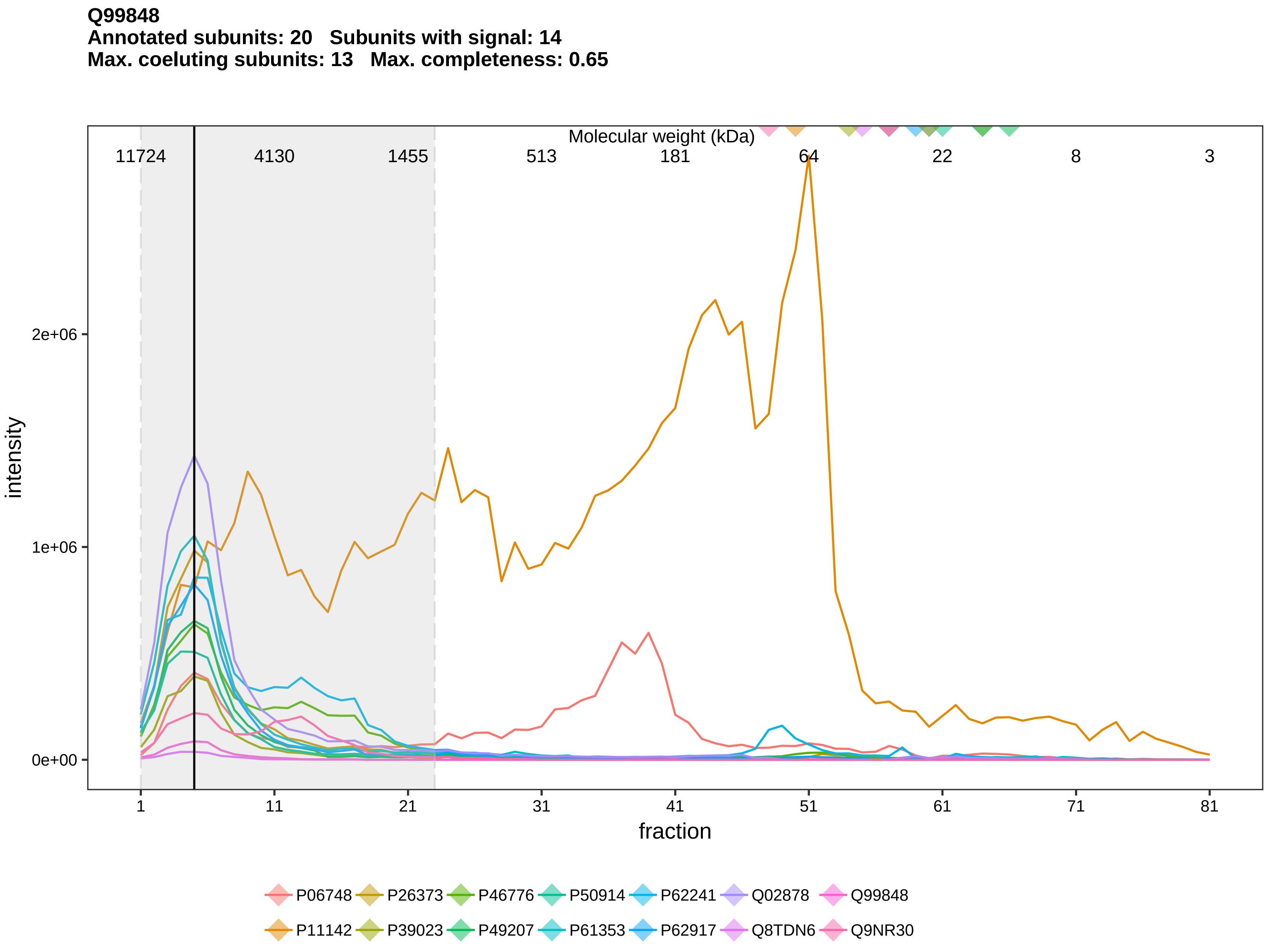
Q99848
Annotated subunits: 20   Subunits with signal: 14
Max. coeluting subunits: 13   Max. completeness: 0.65
Molecular weight (kDa)
11724
4130
1455
513
181
64
22
8
3
intensity
2e+06
1e+06
0e+00
1
11
21
31
41
51
61
71
81
fraction
P06748
P26373
P46776
P50914
P62241
Q02878
Q99848
P11142
P39023
P49207
P61353
P62917
Q8TDN6
Q9NR30
